# The guiding principles for populational COVID-19 vaccine selection: A normative analysis through comparison of the strategies in Hong Kong and Singapore

**DOI:** 10.7189/jogh.12.03004

**Published:** 2022-02-26

**Authors:** Tak Kwong Chan

**Affiliations:** Hong Kong (no affiliation)

COVID-19 vaccination campaigns are under way in many parts of the world to protect individuals, reduce overall infection and ultimately restore normality. There is no doubt control of pandemic is dependent not just on vaccination but also non-pharmaceutical intervention such as social distancing, hand washing and face masking [1]. This said, mass vaccination remains a major tool to combat the pandemic. As with all important public health policies, the vaccination campaign should be outcome driven. A strategy with highest odd of producing the desired outcome should be adopted. Many countries use more than one vaccine, some employing only highly efficacious vaccines while others rely on vaccines of different technologies and efficacies [2,3]. For instance, Hong Kong has been using two vaccines of significantly different technologies and efficacies since the beginning of the vaccination campaign, whereas Singapore recommends two vaccines of same technology and similarly high efficacy. My article aims to normatively derive the guiding principles for populational vaccine selection using comparison of the strategies in Hong Kong and Singapore. These two cities are chosen as they represent two major streams of selection strategies and they each mainly utilise only two vaccines allowing simpler comparison.

## ISSUES

I briefly describe the vaccination campaigns in both cities and then normatively derive the guiding principles for vaccine selection by an inquisitive and comparative approach. Specifically, both cities acknowledge that the vaccination campaigns aim at protecting individuals against infection, safeguarding public health and restoring normality. The analysis compares the vaccine selection strategies in Hong Kong and Singapore and inquisitively questions which strategy better serves social interest of reducing overall infection and restoring normality, which strategy better serves the individual medical interests of the vaccine recipients and whether the efficacy-safety profiles of different approved vaccines should be compared. Finally, the guiding principles for populational vaccine selection are delineated.

## ANALYSES

### Vaccination campaigns in Hong Kong and Singapore

#### Different vaccine selection strategies

Two vaccines of different technologies and efficacies, namely Comirnaty (produced by Pfizer-BioNTech) and CoronaVac (produced by Sinovac BioTech), are being provided in Hong Kong [4]. Singapore recommends two vaccines, Comirnaty (produced by Pfizer-BioNTech) and Spikevax (produced by Moderna), both of which utilise the same mRNA technology; and recommends use of CoronaVac only by persons medically ineligible to use mRNA vaccines in view of its lower efficacy [5]. In both cities, each vaccination centre provides one type of vaccine. Through an online booking system, eligible vaccine recipients can make an appointment for vaccination at a location of their preference.

#### Efficacies of vaccines

Comirnaty and Spikevax use relatively new messenger ribonucleic acid technology (mRNA), as opposed to CoronaVac which is a traditional inactivated vaccine. For Comirnaty, the Phase 3 placebo-controlled study involving around 44 000 subjects showed that after two doses of vaccination, there was a 95% reduction (95% confidence interval (CI) = 90 to 98) ‘in the number of symptomatic COVID-19 cases in the subjects who received the vaccine (8 cases out of 18 198 got COVID-19 symptoms) compared with the subjects who received the placebo (162 cases out of 18 325 got COVID-19 symptoms)’ [6,7]. Spikevax uses the same mRNA technology, with 94% efficacy (95% CI = 89 to 97%), which is comparable to that of Comirnaty [8]. For CoronaVac, Hong Kong policymakers considered the results of a placebo-controlled study involving around 12 000 subjects in Brazil at the time of approving its use. After two doses of vaccination, there was a 51% reduction (95% CI = 36 to 62) ‘in the number of symptomatic COVID-19 cases in the subjects who received the vaccine (85 cases out of 4953 got COVID-19 symptoms) compared with the subjects who received the placebo (168 cases out of 4870 got COVID-19 symptoms)’ [9]. Although CoronaVac meets the minimum efficacy criteria for World Health Organization Emergency Use Listing assessment [10], the overall efficacy of Comirnaty and Spikevax is significantly higher than that of CoronaVac.

**Figure:**
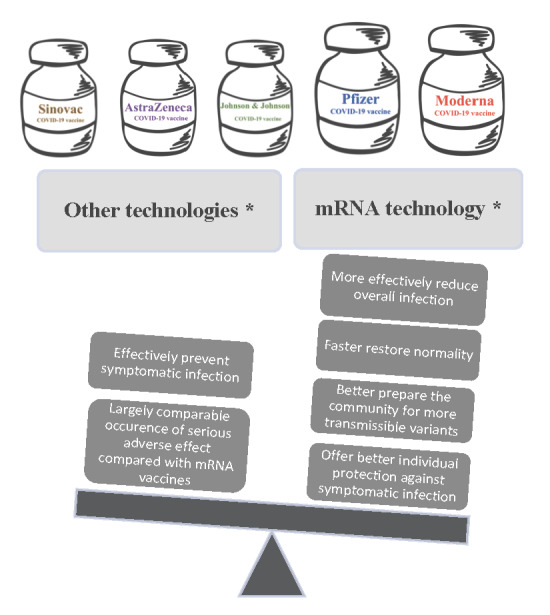
Photo: Comparison between Comirnaty (representing mRNA technology) and CoronaVac (representing other technologies). Based on the generic graphic purchased from https://www.istockphoto.com.

#### Reducing infection and restoring normality

Members of the public are expecting resumption of normal life following mass vaccination. Normality in this context implies withdrawal of harsh restrictive measures (such as quarantine), with return to pre-2020 life, save for some much less disruptive measures such as face masking. Such a vision would be ideally brought about by herd immunity in the city. In order to achieve herd immunity, the required proportion of vaccinated population depends on the efficacy of the vaccine as well as the reproduction number of the virus [11]. For example, to prevent an epidemic, the vaccine needs an efficacy of at least 80% assuming 75% vaccine coverage and reproduction number of 3.5 [11]. Combatting new variants of virus having higher reproduction number will require even higher vaccine efficacy and/or coverage [12].

The advantages to preferentially providing mRNA vaccines are obvious. First, the required covered population would be less for their higher vaccine efficacy. Put another way, herd immunity can be achieved faster. Second, in the process of vaccine uptake pending herd immunity, vaccines of higher efficacy could reduce more cases at possible subsequent peaks, permitting less use of highly restrictive measures such as ‘suppress and lift strategy’ and ‘strict quarantine’ [11]. Last but not least, vaccines of higher efficacy better prepare the community to fight potential future variants of the virus having higher reproduction numbers.

Although reducing symptomatic infection may not necessarily reduce viral transmission, there is increasing emergence of data suggesting that COVID-19 vaccines can help reduce transmission. The mass vaccination campaign in Israel showed estimated effectiveness of 90% (95% CI = 83 to 94) against asymptomatic infection [13]. Another study showed that the likelihood of household transmission was approximately 40 to 50% lower in households of index patients who had been vaccinated 21 days or more before testing positive than in households of unvaccinated index patients [14]. Limitations of these studies granted, it can be reasonably assumed that the vaccines can reduce transmission. In short, vaccines of higher efficacy would likely be more capable of reducing transmission.

#### Considering individual medical interests

Vaccines with higher efficacy in general offer greater protection to recipients. This said, some vaccine recipients may express worry about the safety profile of relatively new mRNA technology. Therefore, the efficacy-safety profiles of vaccines should be fully evaluated when policymakers select vaccines, particularly when vaccines of different technologies are being considered. As shown in **Table 1**, mRNA vaccines (Comirnaty as an example) prove to be superior to vaccines of traditional technologies (CoronaVac as an example) in terms of efficacy overall, for severe disease, for elderly people and for people at risk with comorbidities or obesity, and possibly against mutant viruses [15,16]. In the meantime, there is no evidence of any statistically significant difference between Comirnaty and CoronaVac in the overall incidence of serious adverse effects, and specifically of anaphylaxis, nerve palsy or death. Hence it should be concluded that Comirnaty currently demonstrates a better efficacy-safety profile compared with CoronaVac. The evaluation of the efficacy-safety profile of vaccines should be an on-going process, regularly reviewing post-market data such as adverse effects, duration of the protection, efficacy against new variants, antibody levels, whether and how booster dose needs to be given and so on.

**Table 1:** Comparing the efficacy-safety profiles of mRNA vaccines (Comirnaty as an example) and vaccines of traditional technologies (CoronaVac as an example)

	Comirnaty	CoronaVac
**Efficacies profile**		
Overall	95% (95% CI = 90 to 98) [6]	51% (95% CI = 36 to 62) [9]
Against severe disease	From clinical trial: 75% (95% CI = -153 to 100) [6]	100% (95% CI = 17 to 100) [9]
	(did not meet the prespecified success criterion for statistical analysis due to very low number of severe cases. No conclusion can be drawn) [10]	(did not meet the prespecified success criterion for statistical analysis due to very low number of severe cases. No conclusion can be drawn) [10]
	From mass vaccination in Israel: 92% (95% CI = 75 to 100) [13]	
For elderly people	age ≥65 years: 95% (95% CI = 67 to 100) [6]	age ≥60 years: 51% (95% = -167 to 91) [9]
		(did not meet the prespecified success criterion for statistical analysis due to very limited number of elderly subjects. No conclusion can be drawn) [10]
	age ≥55 years: 94% (95% CI = 81 to 99) [6]	
For people at risk with comorbidities or obesity‡	95% (95% CI = 88 to 99) [6]	Data not available [9]
Against mutant viruses	Preliminary study showed similar efficacy to neutralise mutant viruses that emerged from the UK and South Africa [15]	Data not available [16]
**Summary on efficacies:**		
**Comirnaty proves to be superior to CoronaVac in terms of efficacy overall, for severe disease, for elderly people and for people at risk with comorbidities or obesity, and possibly against mutant viruses from the UK and South Africa**		
**Safety profiles:**		
Overall serious adverse effects§	Similar in the vaccine and placebo groups (0.6% and 0.5%, respectively) [6	The number of serious adverse events was limited and the majority of them were not considered related to the vaccine [9]
Specific adverse effect: anaphylaxis	About 11 cases per million doses administered [6]	Data for anaphylaxis not available [9]
		Incidence of hypersensitivity was about 6 per 100 000 doses [9]
Specific adverse effect: nerve palsy	The incidence rate of acute facial paralysis was less than 1 in 1 000 people, consistent with the background rate in the general population [6,17]	The incidence rate of acute facial paralysis and Guillain Barre Syndrome was lower than the corresponding baseline incidence rate [9]
Death	None proven to be directly attributable to the vaccine has been reported [6,7]	The number of death cases was limited and the majority of them were not considered related to the vaccine [9]
**Summary on safety:** **No conclusion can be drawn on any difference of the two vaccines in the incidence of overall serious adverse effects, anaphylaxis, nerve palsy and death**		

CI – confidence interval

‡At risk is defined in the clinical trial of Comirnaty as having at least one of the Charlson Comorbidity Index categories [18] or obesity (body mass index [BMI]≥30 kg/m^2^) [6].

§Serious adverse events were defined as any untoward medical occurrence that resulted in death, was life-threatening, required inpatient hospitalization or prolongation of existing hospitalization, or resulted in persistent disability/incapacity [17].

There are concerns that mRNA vaccines are associated with higher chance of systemic reaction such as headache, fatigue, chills and fever. But this is a sign that the immune response is successfully induced and the majority of these events were mild or moderate in severity, lasting only one day [17]. These self-limiting short term systemic reactions pose no significant health threat to the recipients. With this in mind as well as the better efficacy-safety profiles of mRNA vaccines, population-wide provision of mRNA vaccines instead of traditional ones are in full conformity with the ethical principles of beneficence and non-maleficence. To achieve greater societal good of safeguarding public health, there are strong ethical imperatives on the ground of justice to deny the recipients the choice of a vaccine. If traditional vaccines are concurrently provided, they should only serve as a backup option for those medically unfit to receive mRNA vaccines. In the alternative, if a choice of allowed, recipients should be strongly encouraged to choose mRNA vaccines.

### Should we compare different vaccines?

One may suggest that differences in the efficacies reported from different clinical trials can arise from factors other than effectiveness, including differences in the trial populations, locations, timing and design. A systematic review of randomised controlled trials that directly compare two interventions head-to-head is generally regarded as the highest quality evidence to support health care decisions [19]. For different reasons, however, best-quality evidence may not always be available. The absence of best quality evidence should not absolve policymakers from seeking and relying on other high-quality evidence [19]. So far as vaccines efficacies derived from clinical trial results are being relied on, one can only appear irrational to avoid a comparison of the efficacy-safety profiles of different vaccines using the same data. Upon recognising the limitations, such comparison should serve as valuable reference in the process of policy decision making. To this end, indirect comparison using adjusted method can be undertaken.

For instance, Hong Kong policymakers considered the trial for CoronaVac conducted in Brazil and the trial for Comirnaty conducted in 152 sites worldwide (including Brazil) [6,9]. mRNA vaccines (Comirnaty as an example) demonstrated comparatively higher efficacy for preventing symptomatic COVID-19 infection with calculated Bucher ratio of 0.10 (95% confidence interval: 0.050.21) (See **Table 2**) [20], which is consistent with the qualitative comparison as exemplified in **Table 1****.**

**Table 2:** Indirect comparison of two vaccines using Bucher’s adjusted method

	Clinical trial for Comirnaty		Clinical trial for CoronaVac	
**Placebo**	**Comirnaty**	**Placebo**	**CoronaVac**
Participants having symptomatic COVID-19 infection	162 out of 18325	8 out of 18198	168 out of 4870	85 out of 4953
Relative risk	0.05 (*P* < 0.0001, 95% CI = 0.02 to 0.10)		0.50 (*P* < 0.0001, 95% CI = 0.38 to 0.64)	
Indirect comparison using Bucher’s adjusted method	Relative risk ratio (for Comirnaty) / Relative risk ratio (for CoronaVac) 0.10 (*P* < 0.0001, 95% CI = 0.05 to 0.21)			

CI – confidence interval

### Deriving the guiding principles for vaccine selection

Although some studies published after Hong Kong started the vaccination campaign showed somewhat higher efficacy for CoronaVac, the numbers are still significantly lower than that of Comirnaty [21,22]. In December 2021, 69% of Hong Kong population were given two doses of vaccine. Among them, 37% received CoronaVac, showing overall populational efficacy of 79% (0.37 × 51% + 0.63 × 95%) [4]. Singapore had 87% of their total population given two doses of vaccine, with overall populational efficacy of over 90% [5]. In hindsight, due to on-going development of new variants of the virus, herd immunity may never be achieved. That said, as delta and probably Omicron variants have a much higher reproduction number and current vaccines have lower efficacy against them [12], the much higher populational vaccine efficacy attained in Singapore would mean that it will be better protected against the variants whereas Hong Kong will never achieve herd immunity with the current vaccine selection strategy [23].

Previously, distribution of mRNA vaccines to individual clinics can be challenging due to the storage issues. Following the recommendation from European Medicines Agency to extend the approved storage period of the unopened vial of mRNA vaccines at 2-8°C from five days to one month [24], there is no logistic reason to provide much less efficacious traditional vaccines. To combat the pandemic, as important as the efficacy of the vaccine is public trust in the campaign, which is critically dependent on the perceived principles that guide government decision in vaccine procurement, among other factors [25]. The ethical principles of justice, beneficence and non-maleficence command policymakers to advance overall social and individual medical interests. As I showed in the above analysis, there are strong imperatives for policymakers to procure vaccines of best efficacy-safety profiles for the population. Missing out on the abovementioned important ethical principles, or putting in irrelevant consideration, would only weaken public trust in the vaccination campaign thereby slowing down the return to normality.

**Figure 1:**
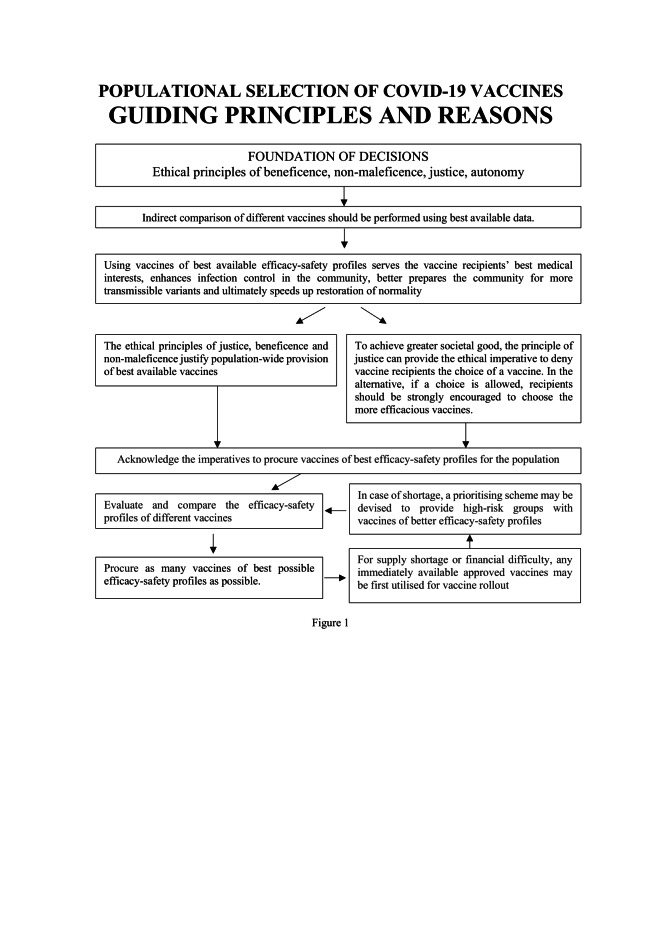
Populational selection of COVID-19 vaccines: guiding principles and reasons.

The guiding principles for populational vaccine selection derived from the above analyses are shown in **Figure 1****.** The starting point is to acknowledge the imperatives to compare vaccines and procure those of best efficacy-safety profiles for the population. Due to global vaccine scarcity, there could be difficulty acquiring sufficient quantities of vaccines with best efficacy-safety profiles even for high-income nations. There may also be concerns for costs for middle and low-income nations. Acknowledging these limitations, immediately available approved vaccines may be utilised for vaccine rollout rather than holding out for better vaccines. In case of shortage, a prioritising scheme may also be devised to provide high-risk groups with vaccines of better efficacy-safety profiles. In parallel, on-going efforts should be made to obtain as many vaccines of best efficacy-safety profiles as possible. Last but not least, despite the primary duty to protect their own citizens, high-income nations should also support global initiatives to promote equitable access to efficacious vaccines in middle and low-income nations.

## CONCLUSIONS

It cannot be emphasised enough that the success of pandemic control depends not just on the rollout of vaccination but also non-pharmaceutical interventions such as hand washing, social distancing and face masking. However, had it not been for the new mRNA technology, the return to normality would be further distant. Policymakers should be prompt to act on indirect comparison of different vaccines with best available data. Using vaccines with better efficacy-safety profiles not only enhances infection control, better prepares the community for more transmissible variants, speeds up restoration of normality, but also serves vaccine recipients’ best medical interests. Applying relevant ethical principles and adopting an outcome-driven approach is essential to fostering public support of vaccination as an important tool to tackle the pandemic. This normative analysis provides a rationalised framework of populational vaccine selection for policymakers all over the world. To sum up my call: while any first available efficacious COVID-19 vaccines may be utilised in vaccine rollout, there should be continuous efforts to procure vaccines of best possible efficacy-safety profiles for the population.
